# Establishment of a 5-gene risk model related to regulatory T cells for predicting gastric cancer prognosis

**DOI:** 10.1186/s12935-020-01502-6

**Published:** 2020-09-03

**Authors:** Gang Hu, Ningjie Sun, Jiansong Jiang, Xiansheng Chen

**Affiliations:** Department of Gastrointestinal Surgery, Yiwu Central Hospital, 699# Jiangdong Road, Jiangdong Street, 322000 Jinhua, China

**Keywords:** Gastric cancer, Regulatory T cells, mRNA signature, TCGA, Prognosis prediction

## Abstract

**Background:**

Gastric cancer (GC) is one of the high-risk cancers that lacks effective methods for prognosis prediction. Therefore, we searched for immune cells related to the prognosis of GC and studied the role of related genes in GC prognosis.

**Methods:**

In this study, we collected the mRNA data of GC from The Cancer Genome Atlas (TCGA) database and studied the immune cells that were closely related to the prognosis of GC. Spearman correlation analysis was performed to show the association between immune cell-related genes and the differentially expressed genes (DEGs) of GC. Univariate and multivariate Cox regression analyses were conducted on the immune cell-related genes with a high correlation with GC. A prognostic risk score model was constructed and the most significant feature genes were identified. Kaplan–Meier method was then used to compare the overall survival (OS) of patients with high-risk and low-risk, and receiver operating characteristic (ROC) analysis was used to assess the accuracy of the risk model. In addition, GC patients were grouped according to the median expression of the features genes, and survival analysis was further carried out.

**Results:**

It was noted that regulatory T cells (Tregs) were significantly correlated with the prognosis of GC, and 172 genes related to Tregs were found to be closely associated with GC. An optimal prognostic risk model was constructed, and a 5-gene (including LRFN4, ADAMTS12, MCEMP1, HP and MUC15) signature-based risk score was established. Survival analysis showed significant difference in OS between low-risk and high-risk samples. ROC analysis results indicated that the risk model had a high accuracy for the prognosis prediction of samples (AUC = 0.717). The results of survival analysis on each feature gene based on expression levels were consistent with the results of multivariate Cox analysis for predicting the risk rate of the 5 genes.

**Conclusion:**

These results proved that the 5-gene signature-based risk score could be used to predict the survival of GC patients, and these 5 genes were closely related to Tregs. These findings are of great significance for studying the role of immune cells and related immune factors in regulating the prognosis of GC.

## Background

Gastric cancer (GC) is a world-class malignant tumor associated with digestive system [1–3]. The 5-year survival rate of patients with GC is generally less than 10% [4]. Patients diagnosed with GC are usually at an advanced stage and there is still no effective treatment for advanced patients [5–7]. In order to find an effective treatment for GC, it is very important to study the potential molecular mechanisms.

Tumor immunotherapy is a new field of oncology research that provides a new therapeutic method for different types of tumors with metastatic characteristics [8–11]. Infiltrating immune cells seem to be the most likely target cells to improve clinical prediction and therapeutic effects [12–15]. Recent experiments of drugs acting on immunomodulatory pathways have shown that when disease-related immune pathways are modulated, they will regulate immune response of patients, which will extend the survival time of patients with a variety of cancers, including melanoma, non-small cell lung cancer (NSCLC), renal cell cancer and GC [13, 16]. Some immune factors, such as PD-L1 and tumor-infiltrating lymphocytes, have been proved to have significant immunological effects on GC [17–19]. However, due to technical limitations, research on immunotherapy for cancer including GC, has been limited to a few types of immune cells. Therefore, analyzing and searching for immune cells and immune cytokines associated with the prognosis of GC is the key of immunotherapy.

According to the principle of “early diagnosis and early treatment”, many methods for early diagnosis or prediction and research on molecular markers have been concerned. In recent studies, many oncogenes or suppressor genes related to GC have been reported. Ba et al. found that EIF5A2 promotes the proliferation and metastasis of GC cells, while thermo-chemotherapy can inhibit GC cell proliferation and metastasis by suppressing EIF5A2 expression [20]. Du et al. demonstrated that APC gene with high expression is a biomarker for poor prognosis in patients with stage T4 GC [21]. Although a large number of genes for prognosis and diagnosis of GC have been discovered, the genes and the multi-gene signatures used for prognosis prediction still need to be studied.

In this study, we downloaded the mRNA data of GC from the TCGA-STAD dataset to find immune cells and genes most relevant to the prognosis of GC. Based on these genes, the feature genes most related to the risk of GC were screened and a multivariate Cox regression model was constructed. The model was also used to assess the survival condition of patients with high-risk and low-risk GC. These results can provide a new reference for the prognosis of GC patients.

## Methods and materials

### Searching for publicly available data from TCGA database

TCGA-STAD mRNA expression data and clinical data were downloaded from the TCGA database (https://www.cancer.gov/about-nci/organization/ccg/research/structural-genomics/tcga) by July 15, 2019, containing 407 samples (375 tumor samples, 32 normal samples).

### CIBERSORT data transformation and differential analysis

The mRNA FPKM data of 407 samples were used to calculate the infiltration abundance of 22 immune cells in each sample using CIBERSORT algorithm. Then, the infiltration levels were clustered. Samples were classified into the Normal group and the Tumor group, and the infiltration levels of the 22 immune cells in two groups were evaluated. Survival analysis was performed on the immune cells with significant differences in infiltration level between the Normal group and the Tumor group. At the same time, the mRNA FPKM data were treated by differential analysis using edgeR (https://bioconductor.org/packages/release/bioc/html/edgeR.html) to obtain differentially expressed mRNAs (DEmRNAs) of GC.

### Spearman correlation analysis and enrichment analysis

Spearman correlation analysis was performed on the regulatory T cells (Tregs) and DEmRNAs to screen genes with a relatively high correlation (*P *< 0.01, |R| > 0.15) as related genes of Tregs. Then, GO and KEGG analyses were performed on these genes.

### Construction of a prognostic multivariate Cox regression model and identification of feature genes

R package “Survival” was used to conduct univariate and multivariate Cox regression analyses on previously selected genes related to Tregs, and an optimal prognostic multivariate Cox regression model was constructed to find independent prognostic factors following the program guidance using default setting. The identified prognostic factors in the risk model were validated in various aspects: (1) The patients were divided into high-risk and low-risk groups according to the median risk value of all samples. Kaplan–Meier method was used to compare the overall survival (OS) of the two groups, and the log-rank method was used for statistical analysis. (2) Receiver operating characteristic (ROC) curves were plotted using the “survivalROC” package and the area under curve (AUC) was calculated.

## Results

### Significant differences in the infiltration level of various immune cells between GC tumor and normal tissues

**Fig. 1:**
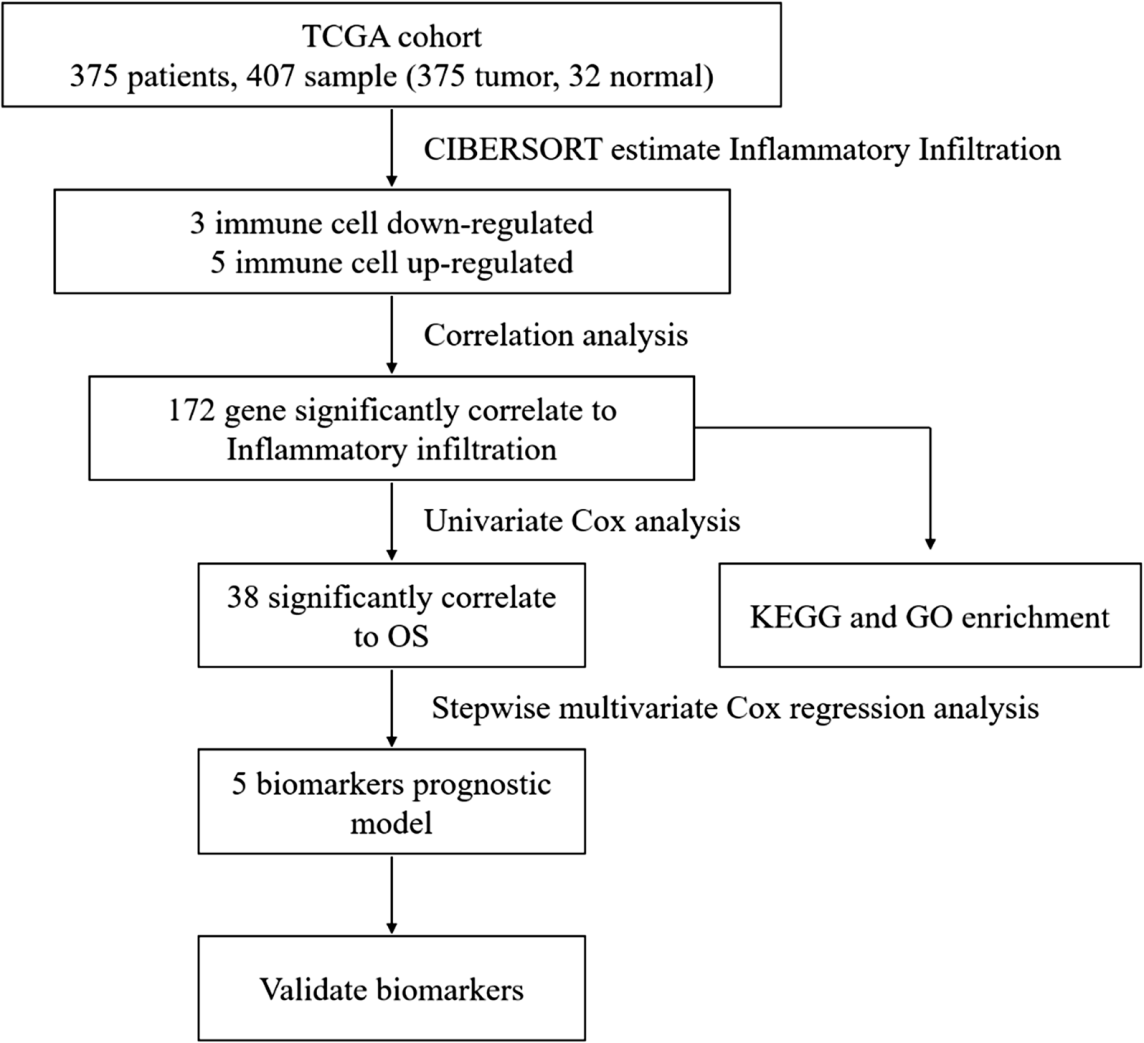
Overview flowchart of this study

**Fig. 2:**
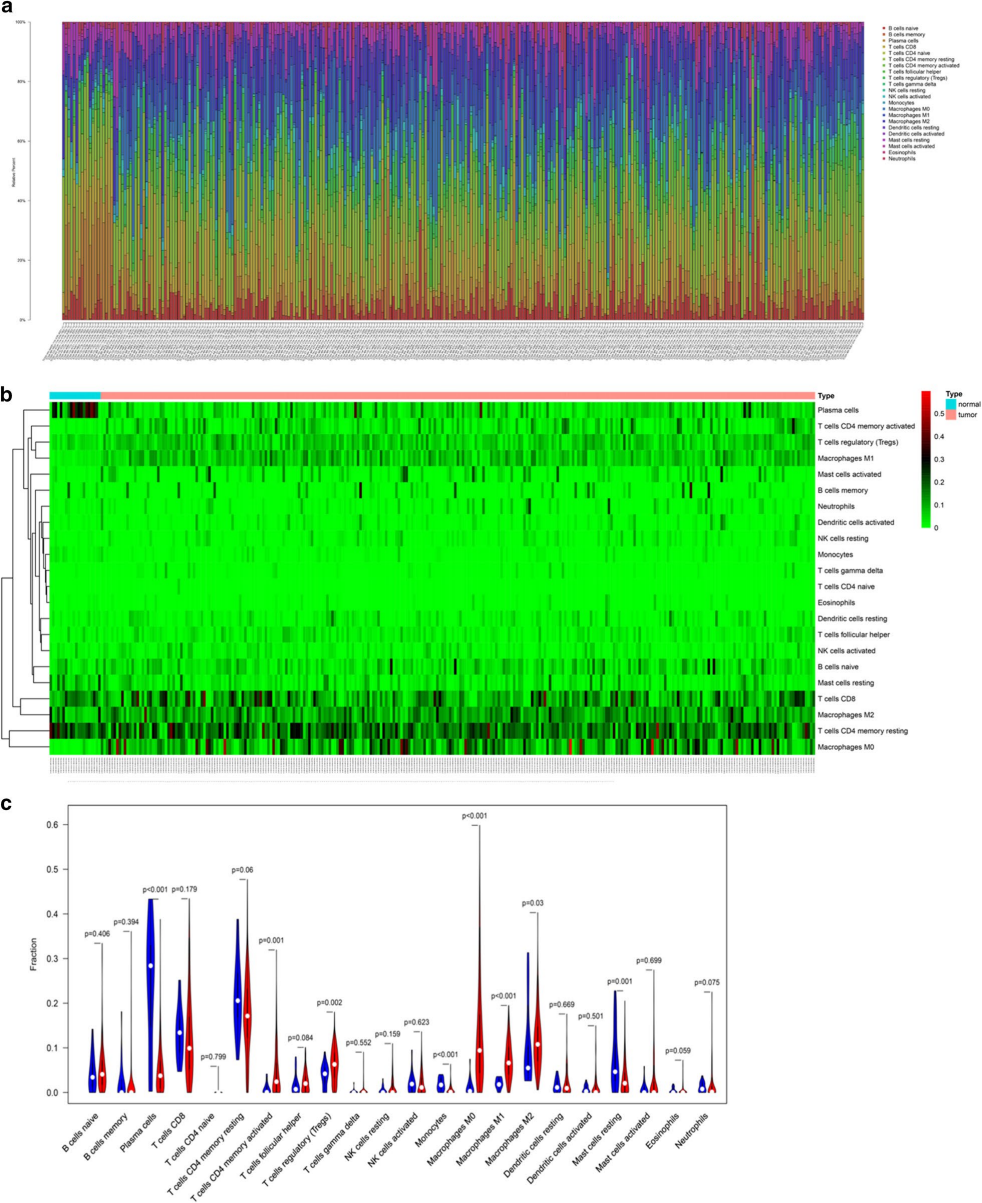
Significant differences in the infiltration level of various immune cells between GC and normal tissues **a** Infiltration abundance of 22 immune cells in each sample; **b** The heat map of the infiltration abundance of 22 immune cells in Tumor group (pink) and Normal group (blue). In the matrix, green to red indicates the level of infiltration gradually rises; **c** The difference in the infiltration level of 22 immune cells in the Normal group and the Tumor group. The blue line represents the Normal group, and the red line represents the Tumor group

To describe our study more clearly, a flowchart of this study was presented (Fig. 1). In order to study the differences in the infiltration level of immune cells between GC tumor and normal tissues, mRNA expression data from 407 GC samples in TCGA-STAD dataset were used to generate infiltration levels of 22 major immune cells in each sample using the CIBERSORT algorithm (Fig. 2a). The data were processed by clustering analysis and the samples were divided into Normal group and Tumor group to compare the infiltration level (Fig. 2b). The results showed that there was no significant difference in infiltration level of most immune cells between the Normal group and the Tumor group. Among the cells with significant difference in infiltration level between the two groups, plasma cells, monocytes and resting mast cells showed a great decrease in the Tumor group (Fig. 2c). The infiltration levels of activated CD4 memory T cells, Tregs, M0 macrophages, M1 macrophages and M2 macrophages in Tumor group were significantly increased compared with those in the Normal group (Fig. 2c). These results indicated that the change in the infiltration level of some immune cells in tumor tissues may have a certain regulatory effect on tumorigenesis.

### Enrichment analysis of Tregs-related mRNAs

**Fig. 3:**
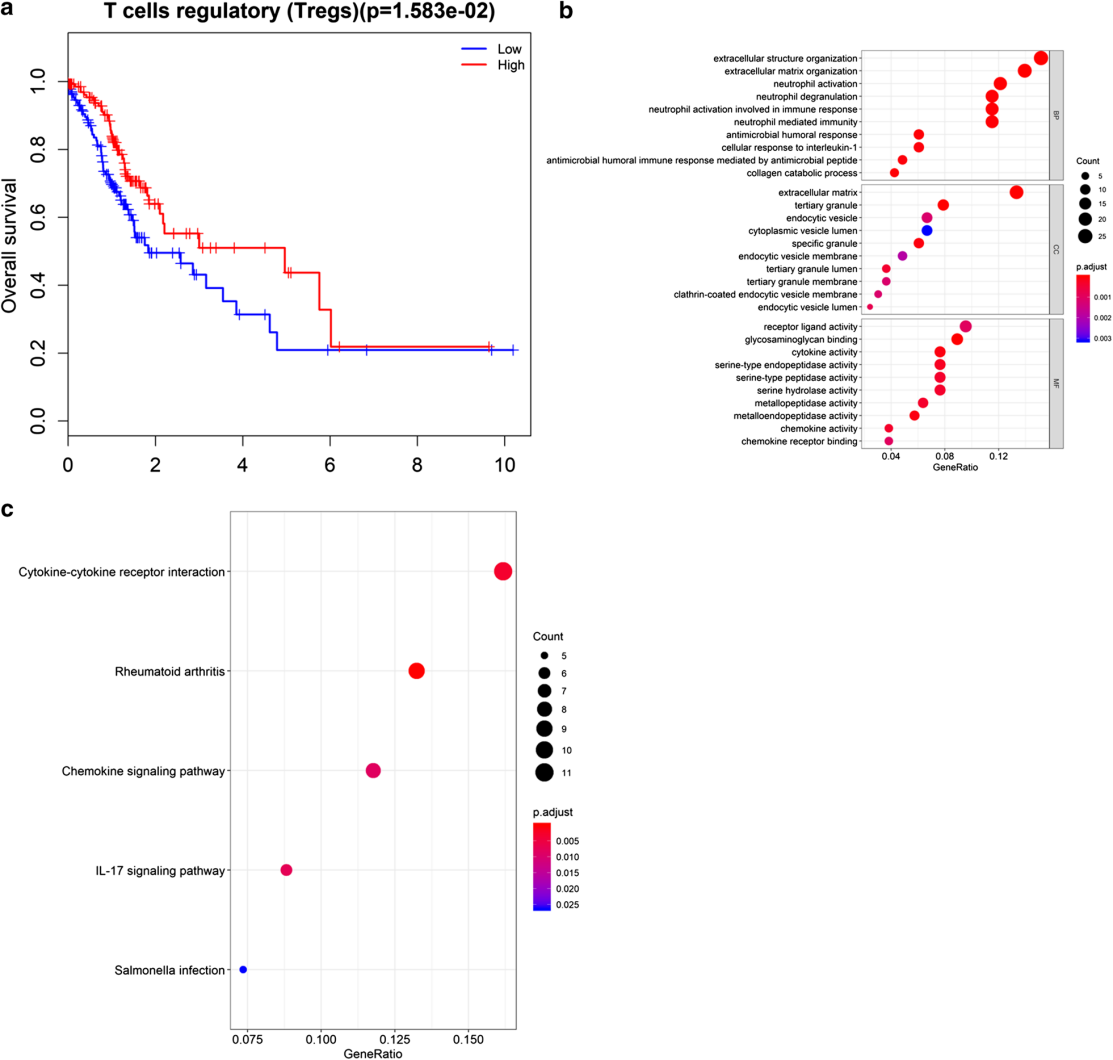
Enrichment analyses of Tregs-related mRNAs **a** Survival curves show the effect of infiltration level of Tregs on OS of patients. The blue line represents low infiltration, red line represents high infiltration; **b** GO enrichment analysis of Tregs-related genes; **c** KEGG enrichment analysis of Tregs-related genes

In order to study the immune cells closely related to the prognosis of GC and their related mRNAs, survival analysis was performed on 8 immune cells with significant differences in infiltration level in the Tumor group and the Normal group. The result exhibited that only the patients with a high or low infiltration level of Tregs had significant difference in OS (Fig. 3a). OS of patients with high infiltration was significantly longer than that with low infiltration, so Tregs were selected for further study. Spearman correlation analysis was performed on Tregs and DEmRNAs and 172 genes with a high correlation were screened (*P *< 0.01, |R| > 0.15) (Additional file 1: Table  S1). GO enrichment analysis of the 172 genes revealed that these genes were enriched in a large number of immune-related biological functions, such as neutrophil activation, neutrophil degranulation and tertiary granule (Fig. 3b). The KEGG analysis also showed that most of these genes were enriched in two immune-related pathways, Rheumantoid arthritis and IL-17 signaling pathway (Fig. 3c). Therefore, Tregs may play an important role in regulating GC, and its related genes may also affect the prognosis of GC.

### Establishment and evaluation of a prognostic risk model

**Fig. 4:**
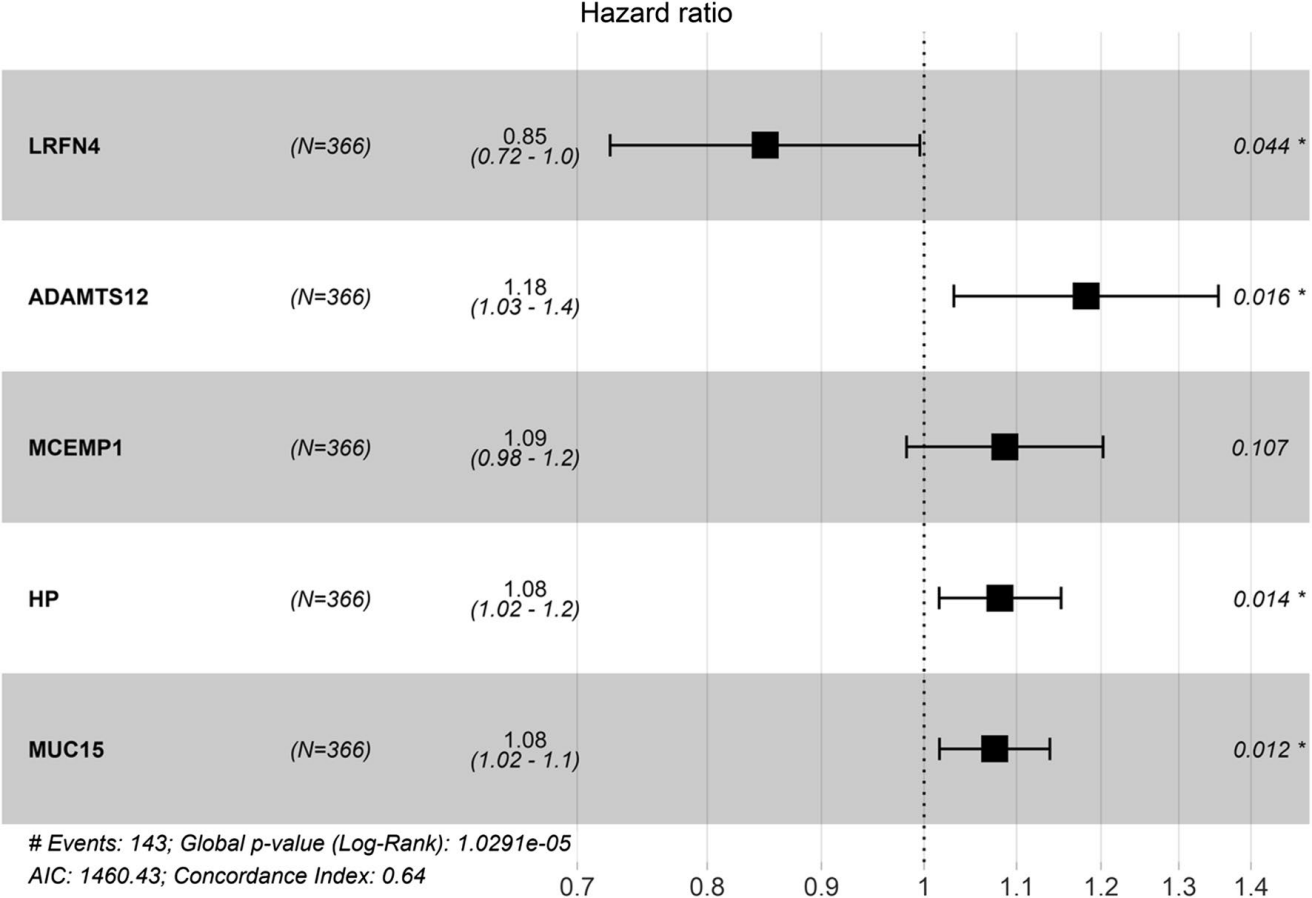
Forest map of multivariate regression analysis

In order to comprehensively investigate the relationship between Tregs-related genes and prognosis of GC, the 172 Tregs-related genes were used for univariate regression analysis and 38 genes with *p *< 0.05 were screened (Additional file 1: Table S2). These 38 genes were then used for stepwise multivariate Cox regression analysis to construct a prognostic risk model according to the program guidance. Finally, five genes (LRFN4, ADAMTS12, MCEMP1, HP, MUC15) were identified that could be used as independent prognostic factors for GC (Fig. 4). Among the 5 genes, LRFN4 was seen to have lower risk (Hazard ratio < 1) while the other 4 genes had higher risk (Hazard ratio > 1). Then, a 5-gene signature-based risk scoring formula was constructed to evaluate the survival risk of the samples according to the expression levels of the 5 genes: risk score = (–0.1634 × expression level of LRFN4) + (0.1669 × expression level of ADAMTS12) + (0.083085 × expression level of MCEMP1) + (0.078267 × expression level of HP) + (0.072812 × expression level of MUC15).

**Fig. 5:**
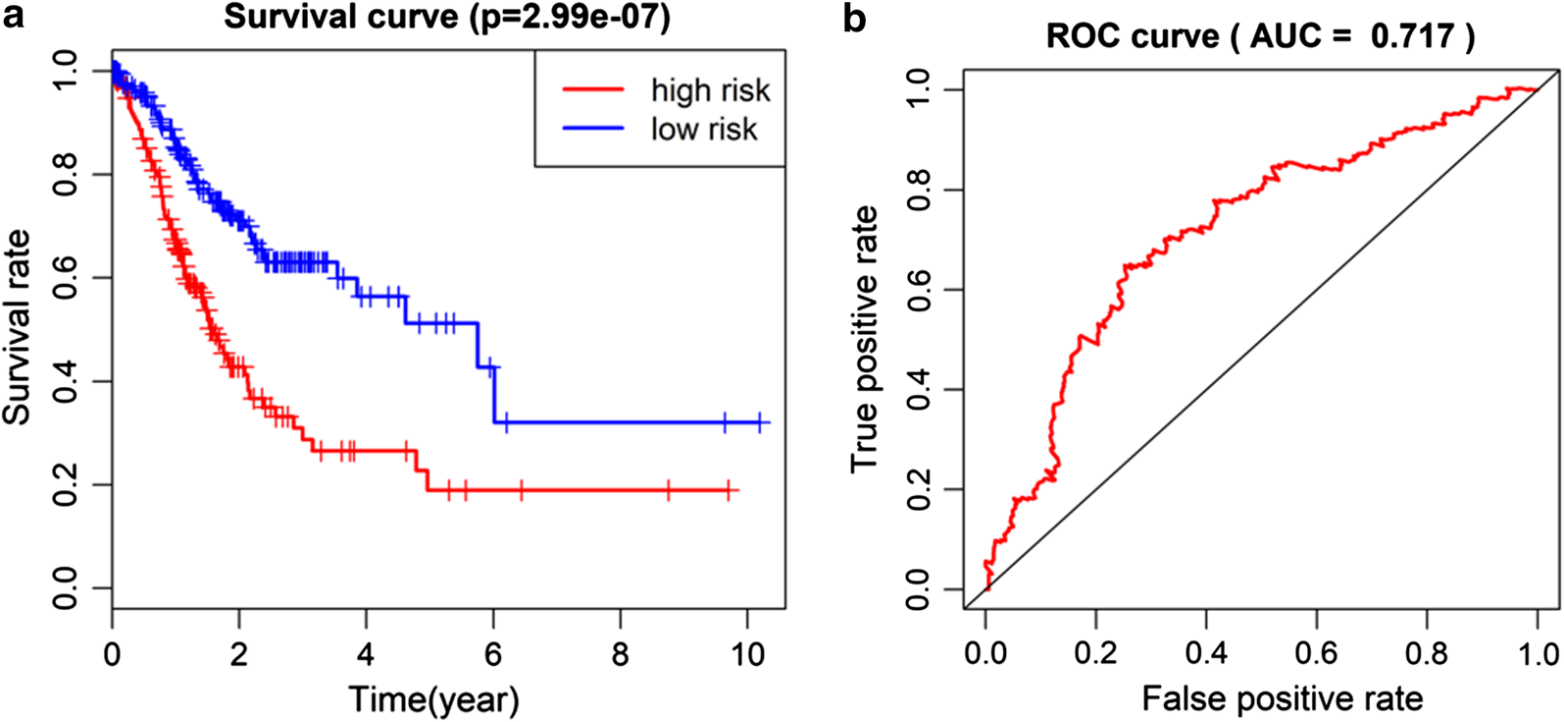
Kaplan–Meier survival curves of patients in high- and low-risk groups and ROC curves of the risk model **a** Kaplan-Meier survival curves show the effect of the 5-gene risk score on OS of patients with GC. The blue line represents low-risk group and the red line represents high-risk group; **b** ROC curves for assessing the sensitivity and specificity of the 5-gene risk score to predict survival risk of patients

After the risk scoring formula was established, we classified each sample according to the formula to assess their survival rate. Patients were divided into high-risk and low-risk groups based on the median risk score. As exhibited in Fig. 5a, OS of the low-risk group was significantly higher than that of the high-risk group. ROC curves were also drawn to compare the sensitivity and specificity of the risk score in survival prediction (Fig. 5b). The results displayed that the AUC value was 0.717, which proved that the risk score had good sensitivity and specificity in prognosis prediction.

### Survival analysis of the 5 feature genes

**Fig. 6:**
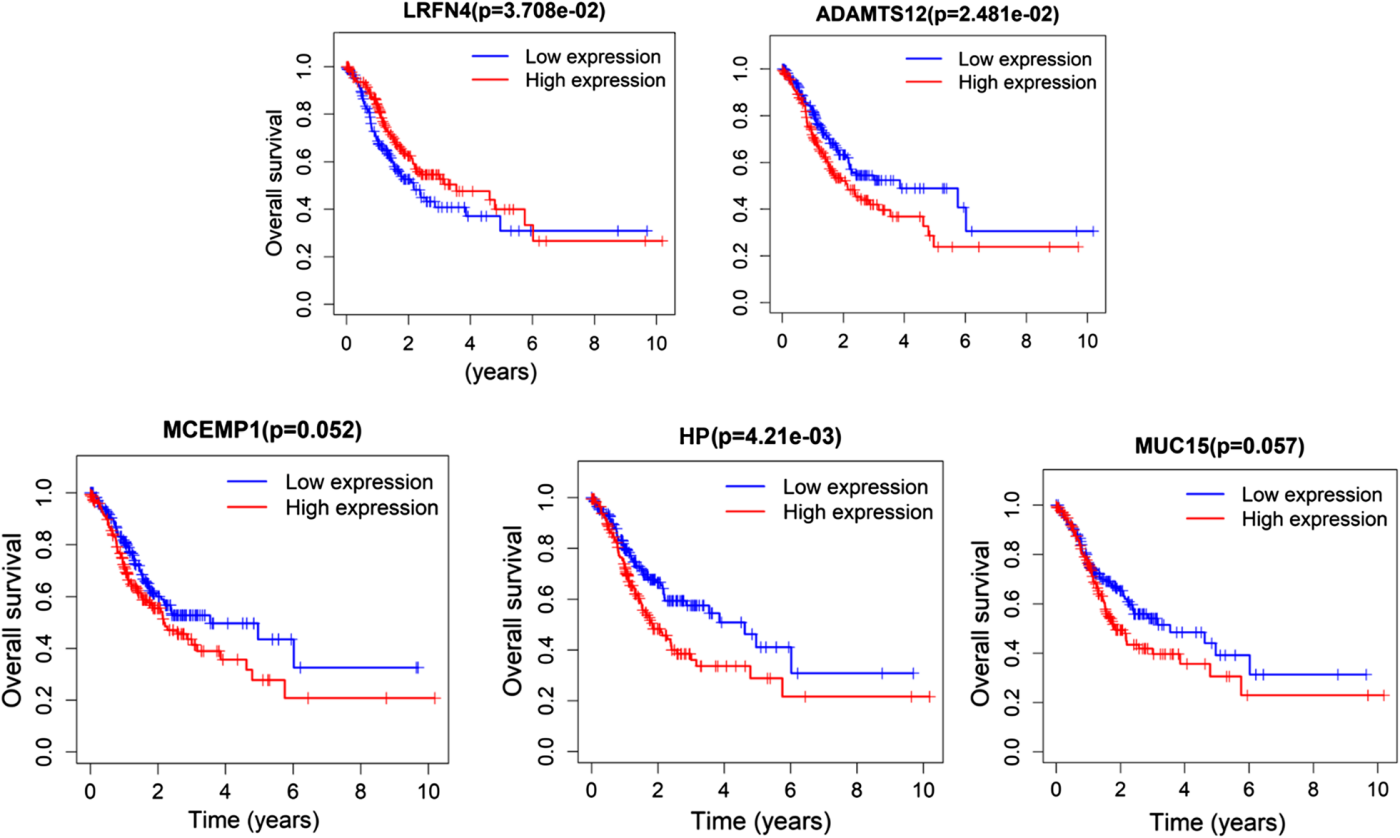
Kaplan-Meier survival analysis of 5 feature genes Red represents high gene expression and blue represents low gene expression

In order to verify the accuracy of the risk assessment system, patients were respectively grouped according to the median expression of the five genes, and Kaplan–Meier curves were drawn for survival analysis. The results revealed that the survival rate of patients with high expression of ADAMTS12 and HP was significantly lower than those of patients with low expression. The survival rate of patients with high expression of LRFN4 was remarkably higher than that of patients with low expression (Fig. 6). These results were consistent with the previous conclusions.

## Discussion

In this study, we used the mRNA expression data of GC to analyze and prove that there were 8 immune cells including Tregs presenting significant differences in infiltration level in GC and normal tissues. Among them, only Tregs showed significant differences in the survival rate between the high infiltration and low infiltration groups. Many previous studies reported that Tregs are associated with the development of multiple cancers including GC. Olkhanud et al. reported that tumor-induced regulatory B cells in breast cancer (BC) could transform dormant CD4 + T cells into Tregs and promote the metastasis of BC [22]. Andreas et al. studied the immunoreaction concerning p53 in head and neck cancer (HNC) and found that a certain amount of specific T cells and Tregs are clustered around p53 peptide tetramers [23]. These results suggest that Tregs are closely related to the prognosis of GC.

For purpose of studying the immune factors related to Tregs which regulate the progression of GC, we screened 172 Tregs-related genes that were closely related to GC. An optimal multivariate Cox regression model consisting of 5 genes (including LRFN4, ADAMTS12, MCEMP1, HP and MUC15) was constructed based on the 172 selected genes, and a 5-gene signature-based risk score was established. LRFN4 is regarded as a low-risk gene closely related to low-risk death and survival time prolongation. As a neuronal transmembrane protein, LRFN4 mainly plays a role in human immune system. Konakahara et al. demonstrated that LRFN4 induces monocyte/macrophage migration via actin cytoskeleton reorganization [24]. It is also the first time LRFN4 proved to be associated with GC prognosis. Therefore, the specific effects of LRFN4 on GC and other cancers need further studies. The other four genes were considered as high-risk genes in this study, and they were considered to be closely associated with high-risk death. The MCEMP1 gene is found to be associated with the prognosis of GC for the first time. It was reported that HP expression would be induced by IL-6 through activating STAT3 in HNC [25]. CHoi et al. found that MUC15 promotes the tumorigenesis of thyroid cancer and the growth of stem cells via GPCR/ERK and integrin-FAK signaling pathways [26]. However, ADAMTS12 is considered as a tumor suppressor in other cancers. Wang et al. reported that ADAMTS12 is a favorable prognostic marker in colorectal cancer, and its high expression would inhibit the occurrence of tumors [27]. In view of the differences in the roles of these feature genes in different cancers, we will conduct further studies on the specific roles of these genes in the survival of GC patients in the future.

Prognostic biomarkers refer to biomarkers related to prognosis of cancer patients, and they can be gene expression (mRNA), protein content or small molecular metabolites [28]. As prognostic markers can be independent to determine the prognosis of patients and assist the diagnosis and treatment of cancer in clinical practice, searching for prognostic markers related to cancer prognosis has become a hot research topic [29–31]. Many experiments have been conducted to screen and explore prognostic markers of GC, among which the most famous one is PD-L1 gene. A study has found that PD-L1 is highly expressed in cancer tissue of GC patients, which inhibits anti-tumor immune response and enables tumor cells to escape from the killing of immune cells, showing its role as the most important gene in the immune escape mechanism of cancer cells [32]. Another study has indicated that PD-L1 can be used to predict the prognosis of GC patients, but the evaluation effect is not ideal and only 43% to 63% cancer tissues of Asian cancer patients are positive for PD-L1, and the expression of PD-L1 is decreased in tumor tissues of many sufferers [33]. It is not accurate to use individual gene to judge the prognosis of cancer patients owing to the differences in race, region and organ specificity [33, 34]. In recent years, with the development of computer technology, researchers have begun to use regression models, artificial intelligence and other computational methods to screen and analyze gene expression data in cancer databases on a large scale to explore prognostic biomarkers associated with specific cancers, such as this study [35]. LRFN4, ADAMTS12, MCEMP1, HP and MUC15 are all cancer-related biomarkers, and all of them can also be used in judgment of cancer incidence in an independent manner. Compared to using PD-L1 alone to judge the prognosis of patients, this study screened prognosis-related genes based on TCGA, and the data we analyzed already involved geographical and racial differences, resulting in better applicability and accuracy in cancer diagnosis.

We established a 5-gene risk model based on the feature genes related to the survival of GC, and used it to evaluate the survival risk of the downloaded samples. The survival curves showed that the OS of patients in the high-risk group was significantly shorter than low-risk patients. This risk score further proved the important role of these feature genes in evaluating the survival of patients with GC and also demonstrated the role of Tregs-related genes in the development and prognosis of GC for the first time. Nevertheless, due to epidemiological limitations, we were unable to detect the specific correlation between this simulated risk score and the prognosis of GC. So, verification and study of the model need to be further developed.

In conclusion, we demonstrated that the 5 feature genes related to Tregs were closely associated with the prognosis of GC. This 5-gene risk score model can be used to evaluate the survival risk of patients with GC and provides a basis for studying the role of immune cells and their related factors in the prognosis of GC.

## Supplementary information

**Additional file 1:** **Supplementary Table 1.** 172 genes with high correlation were screened (P <0.01, |R|> 0.15); **Supplementary Table 2.** 172 Tregs related genes were selected for univariate regression analysis and 38 genes with P < 0.05 were screened

## Data Availability

The data used to support the findings of this study are included within the article. The data and materials in the current study are available from the corresponding author on reasonable request.
